# Assessment of complementary and alternative medicine use among patients admitted to the emergency room: a descriptive study from a Turkish hospital

**DOI:** 10.7717/peerj.7584

**Published:** 2019-08-20

**Authors:** Hakan Hakkoymaz, Burhan Fatih Koçyiğit

**Affiliations:** 1 Faculty of Medicine Department of Emergency Medicine, Kahramanmaras Sütcü Imam University, Kahramanmaraş, Turkey; 2 Department of Physical Medicine and Rehabilitation, Kahramanmaras Sütcü Imam University Faculty of Medicine, Kahramanmaras, Turkey

**Keywords:** Complementary medicine, Alternative medicine, Emergency room

## Abstract

**Background:**

The main aim of this study was to assess the frequency of use of complementary and alternative medicine (CAM) in patients admitted to the emergency room (ER). Additionally, we aimed to evaluate the socio-demographic and clinical factors associated with CAM use.****

**Methods:**

This was a descriptive study. A total of 951 patients who were admitted to the ER of a tertiary hospital between October 2018 and November 2018 were enrolled. Data were obtained using a questionnaire that was prepared by the researchers considering the literature data.

**Results:**

The mean age of the patients was 37.98 ± 15.65 years. Of the 951 patients, 48.4% (*n* = 460) were female and 51.6% (*n* = 491) were male. The rate of patients who used CAM at least once was 47.3% (*n* = 450). The most frequently used CAM methods were herbal therapy (68.9%), massage (40.7%), dietary supplements (24.7%), and hijama (24.2%). Being aged 64 years or younger (*p* = 0.001), having an education level of university or higher (*p* = 0.006), having an income more than minimum wage (*p* = 0.016), and having a chronic disease (*p* = 0.003) were found to be associated with CAM use in this study.

**Discussion:**

CAM methods were used by a considerable proportion of patients admitted to the ER. Physicians should incorporate CAM use history in their patient assessments and should provide accurate and unbiased information about CAM methods.

## Introduction

Complementary and alternative medicine (CAM) is not considered a part of conventional medicine, and is defined as “a series of methods outside the dominant healthcare system used in the prevention and treatment of diseases” (Waterbrook et al., 2010). Although the efficacy and adverse effects of CAM methods are still doubtful, the preference rates for these methods have increased worldwide (Koç & Çınarlı, 2018). Factors such as culture, sex, age, race, socioeconomic status, religion, and education level can affect the rate of use of CAM methods (Tindle et al., 2005). In Turkey, the usage rates of up to 87% CAM were reported in studies conducted for different diseases (Tan, Uzun & Akçay, 2004; Akyüz Özdemir, Erdal & Haberal, 2018; Okyay & Koçyiğit, 2018; Özkan, Karaca & Sarak, 2018). The main reasons why people prefer CAM methods are to avoid the adverse effects of drugs, maintain a life without drugs, to strengthen the immune system, the belief that CAM methods are natural and harmless, positive feedback from other patients, and curiosity (Waterbrook et al., 2010). The increasing use of CAM has led health ministries to take steps in this area of medicine. In Turkey, regulations for CAM methods were published in 2014, and their application criteria were determined. In these regulations, the qualifications of practitioners and the necessary education programs for applications were determined (Turkish Official Gazette, 2014).

Herbal therapy, multivitamins, and supplements have been shown to be an important part of CAM methods (Jatau et al., 2016). High rates of herbal therapy use, however, may cause toxicity, and potential reactions, such as herbal therapy–drug, or herbal therapy–disease interactions (Tulunay et al., 2015). Additionally, previous studies have demonstrated a higher prevalence of CAM usage among patients in the emergency room (ER), than in the general population (Kim et al., 2005; Jatau et al., 2016). Therefore, it is important for health service providers to question the usage of CAM during admission to the ER, to prevent probable adverse effects or reactions. In a study, up to 70% of patients admitted to ERs did not report the usage of CAM methods (Jatau et al., 2016). Considering the potential risks of CAM, the importance of questioning the usage of CAM emerges.

Although there are limited studies that evaluated the use of CAM in ERs, public hospitals, or general practice clinics in Turkey, no study has been conducted in our local region (Şimşek et al., 2017; Koç & Çınarlı, 2018). Geographic location, culture, tradition, and the socio-economic structure of an area may influence the usage of CAM. Therefore, we aimed to obtain relevant data in Kahramanmaraş. Our primary aim was to evaluate the frequency of CAM use in patients admitted to the ER. Our second objective was to reveal the most commonly used CAM methods, and also the socio-demographic and clinical factors associated with their use. Finally, we planned to assess the patients’ opinions about CAM methods, their reasons for using these, and the sources of information about these methods.

## Material and Methods

### Study design and participants

This was a descriptive study. Nine hundred fifty-one patients, who were admitted to the ER of Kahramanmaraş Sütcü Imam University Training and Application Hospital, with various diseases or symptoms between October 2018 and November 2018, were enrolled. Kahramanmaraş Sütcü Imam University Training and Application Hospital is the referral center of our region. In the ER unit, different groups of patients are admitted and evaluated. We conducted this study in the ER because the ER is like a mirror, reflecting nearly all patient groups in the local community. Kahramanmaraş has a different structure in terms of culture, tradition, and socioeconomic indices, as compared with other cities in Turkey, where similar studies have been conducted. Kahramanmaraş is located in the east Mediterranean region of Turkey, with a population of around 1 million, which is predominated by the Muslim community. Due to its proximity to the Syrian border, a significant number of Syrian refugees live in this region. Compared with other provinces, Syrian refugees can affect the demographics and culture of Kahramanmaraş. Additionally, refugees have led to a rise in the unemployment rate. Agriculture and industry have an important role in the city’s economy. A large population lives in rural areas because of the agricultural demographics. Therefore, many patients have problems in gaining access to health services, and thus, turn to using traditional treatment options. In Kahramanmaraş, the society is firmly attached to religion and traditions, which have a stronger influence on the way of life of society, compared with other cities in Turkey.

The inclusion criteria for patients in the study were age 18 years or above, and acceptance to participate.

Patients with high levels of pain, major disease and trauma, dementia, suicide, lack of communication skills, communication problems due to language differences, sedation, aphasia, and poor general conditions were excluded from the study.

### Data collection

A questionnaire was prepared by the researchers, considering previous literature, and was used for collecting data (Waterbrook et al., 2010; Şimşek et al., 2017; Koç & Çınarlı, 2018). The questionnaire was filled by 20 volunteer participants for a pretest analysis. Questions that could not be understood by the participants were reviewed and reorganized. Next, the final version of the questionnaire was composed. Face-to-face interview method was used for filling the questionnaire. Data were collected by the researchers using the questionnaire, after the patients had consulted an ER physician. Completion of the questionnaire took approximately 10–15 min per participant. Data quality control was assessed by the researchers using the hospital database, where the patients’ hospital records were cross-checked using the hospital registration system. The questioned CAM methods are described below (Turkish Official Gazette, 2014):

*Acupuncture:* Stimulation of specific points in the body using needles, laser rays or electrical stimulation.

*Apitherapy:* Bees and bee products used as supportive therapy in the treatment of some diseases.

*Herbal therapy:* A treatment modality using traditional herbal medicinal products and herbal medicines.

*Supplements:* One or more dietary components (including vitamins, minerals, amino acids or other substances) are used.

*Hypnosis/meditation:* This method involves a trance-like state in which one has heightened focus and concentration. Hypnosis is usually performed with the help of a therapist using verbal repetition and mental images.

*Homeopathy*: An holistic approach that aims to improve health status with selected homeopathic medicines.

*Chiropractic*: Deals with biomechanical disorders of the muscle, spine, and skeletal system. It focuses on the correction of mechanical immobility of the joints using manual methods.

*Cupping*: Dry cup application based on creating a regional vacuum to increase blood circulation.

*Leech:* The use of leeches in medical treatment. Leeches can be applied to areas of the body such as the finger, hand, toe, leg, ear, nose, or scalp.

*Hijama:* Wet cupping in which blood is taken by creating superficial skin incisions.

*Mesotherapy*: The use of regional or small dosage injections of herbal or other pharmacological agents, aimed at the healing of mesoderm-induced pathologies.

*Prolotherapy*: Injections of proliferative and irritant solutions into the connective tissue of joints. Pharmaceutical mixes are applied regionally using special needles and techniques.

*Osteopathy*: Helps strengthen the musculoskeletal system; focuses on total body health and on the activity of the musculoskeletal system in diseases.

*Ozone*: Use of a mixture of local or systemic ozone-oxygen.

*Reflexology*: This is based on the principle of the presence of directional reflex areas on the entire body, including the hands, feet, and the ears. Pressure is applied to these reflex areas.

*Massage*: Defined as pressing, rubbing, and moving muscles and other soft tissues of the body, using hands and fingers. The primary aim is to increase the flow of blood and oxygen to the targeted area.

*Aromatherapy*: Full or partial body application or inhalation of fragrant essential oils (from flowers and fruits), for therapeutic purposes.

*Music therapy*: Clinical and evidence-based use of music to meet the physical, psychological, and social needs of individuals.

The sociodemographic details of the participants, including age, sex, education level, marital status, income, and social security status were evaluated in the first part of the questionnaire.

In the second part, the clinical features of the participants, such as the reason for admission to the ER, presentation frequency to the ER, presence of chronic disease, and drug use for chronic disease were evaluated.

In the third part, the lifetime practice history of CAM was evaluated. If positive, the methods used were asked. Patients’ opinions about CAM methods, including the reasons for using CAM methods, and the sources of information about them were assessed.

### Ethical statement

Before the interview, the participants were informed that the choice of participation was fully voluntary and that the data obtained from the forms would be used scientifically. The Medical Ethics Committee of Kahramanmaraş Sütcü Imam University approved the study (Decision date: 12.09.2018, Decision number: 14).

### Statistical analysis

Statistical analyses of the data were performed using the Statistical Package for the Social Sciences (IBM SPSS Statistics for Windows, Version 20.0. IBM Corp., Chicago, IL, USA). Mean ± standard deviation, median, numbers, and percentages were used to express the descriptive data. Normality was assessed using the Shapiro–Wilk test. The continuous variables of the two groups were compared using the Mann–Whitney *U* test. The chi-square test was used to determine the differences between the groups for categorical variables. Additionally, binary logistic regression analysis was performed to detect factors that were predictors of CAM use. *P* < 0.05 was considered as statistically significant.

## Results

A total of 951 patients in the ER completed the questionnaire. The mean age of the patients was 37.98 ± 15.65 years. Of the 951 patients, 48.4% (*n* = 460) were female and 51.6% (*n* = 491) were male. The sociodemographic data of CAM users and non-users including age, sex, education level, marital status, and social security status are summarized in Table 1. The percentage of patients who had used CAM at least once was 47.3% (*n* = 450).

**Table 1:** Sociodemographic characteristics of the complementary alternative medicine users and nonusers.

Characteristics	Nonusers (*n* = 501)	CAM users (*n* = 450)	*p*
Age	35 (18–92)^*^	34 (18–89)^*^	0.922
Sex			
Female	252	208	0.209
Male	249	242	
Education			
Literate	42	35	<0.001
Primary school	119	58	
Middle school	50	58	
High school	126	106	
University	164	207	
Marital status			
Married	329	278	0.301
Single	144	150	
Widorved	28	22	
Social security			
Yes^*^	474	427	0.848
No	27	23	

**Notes.**

n, number

* median (minimum-maximum).

The most frequent reasons for admission to the ER were respiratory (19.6%), gastrointestinal (18.5%), and musculoskeletal (17.9%) symptoms. The median admissions to an ER within one year were three. Of the total patients, 34.1% (*n* = 324) had a chronic disease, and 26.5% (*n* = 252) regularly used drugs for chronic diseases (Table 2).

**Table 2:** Distribution of the patients characteristics admission to emergency department.

Characteristics	n (%)	Median (min–max)
Reason for admission		
Musculoskeletal	170 (17.9)	
Gastrointestinal	176 (18.5)	
Respiratory	186 (19.6)	
Cardiovascular	87 (9.1)	
Neurological	73 (7.7)	
Urological	51 (5.4)	
Gynecological	37 (3.9)	
Addiction/psychiatry	13 (1.4)	
Other	158 (16.6)	
Admission frequency in a year		3 (1–80)
Chronic disease		
Yes	324 (34.1)	
No	627 (65.1)	
Chronic drug use		
Yes	252 (26.5)	
No	699 (73.5)	

**Notes.**

n number % percentage min minimum max maximum

Out of all the patients, 18.7% (*n* = 178) reported that they were against CAM and were not interested in their use. However, 50.4% (*n* = 479) of the patients stated that they were not against CAM methods, and 30.9% (*n* = 294) were interested in CAM. The most frequently used CAM methods were herbal therapy (68.9%), massage (40.7%), dietary supplements (24.7%), and hijama (24.2%). These data are summarized in Table 3.

**Table 3:** Type of complementary and alternative medicine used.

CAM type^*^	n (%)
Acupuncture	13 (2.9)
Apitherapy	11 (2.4)
Herbal	310 (68.9)
Supplements	111 (24.7)
Hypnosis/meditation	6 (1.3)
Homeopathy	0 (0)
Chiropractic	27 (6)
Cupping	31 (6.9)
Leech	34 (7.6)
Hijama	109 (24.2)
Mesotherapy	8 (1.8)
Prolotherapy	0 (0)
Osteopathy	0 (0)
Ozone	2 (0.4)
Reflexology	0 (0)
Massage	183 (40.7)
Aromatherapy	1 (0.2)
Music therapy	42 (9.3)
Other	24 (5.3)

**Notes.**

n number % percentage

* Multiple answers, total does not add to 100%.

Of the CAM users, 17.9% (*n* = 79) reported that a healthcare professional had performed the CAM procedure on them. Additionally, 49.8% (*n* = 24) completely benefitted and 45.3% (*n* = 204) partially benefited from the CAM procedure.

We found that 60.2% (*n* = 271) of patients used CAM methods because they had received positive advice about them; 40.4% (*n* = 182), to avoid drug adverse effects; 19.8% (*n* = 89) just to try them; 16% (*n* = 72), to strengthen the immune system; 15.8% (*n* = 71), because the conventional medicine that they had used was inadequate; 13.1% (*n* = 59), because a healthcare professional recommended it; and 4% (*n* = 18), because CAM methods were cheaper. The information sources of the patients were relatives (71.6%), the Internet (40.4%), media (36.7%), healthcare professionals (24.9%), and books/journals (11.6%) (Table 4).

**Table 4:** Comments and perceptions of the CAM users.

Characteristics	n (%)
**Was the CAM method performed by a health care personnel?**	
Yes	79 (17.6)
No	371 (82.4)
**Did you benefit from the CAM method?**	
Yes	224 (49.8)
Partially	204 (45.3)
No	22 (4.9)
Reason for using CAM method^*^	
. Since a health care personnel suggested	59 (13.1)
. Since conventional medicine was inadequate	71 (15.8)
. To avoid drug side effects	182 (40.4)
. To strengthen the immune system	72 (16)
. Since others tried, and gave good advice	271 (60.2)
. To try	89 (19.8)
. Since CAM methods are cheaper	18 (4)
Information source of CAM^*^	
- Health care personnel	112 (24.9)
- Media	165 (36.7)
- Internet	182 (40.4)
- Relatives	322 (71.6)
- Book/journal	52 (11.6)

**Notes.**

CAM complementary and althernative medicine n number % percentage

* Multiple answers, total does not add to 100%.

Binary logistic regression analysis was performed to evaluate the factors that affected CAM use. Sociodemographic and clinical variables that might affect the use of CAM were added to our study model by considering literature data and similar studies (Gözüm, Tezel & Koc, 2003; Kim et al., 2005; Yates, Armour & Pena, 2009; Jatau et al., 2016). The ‘enter’ method was used in the analysis and collinearity was checked. The participants were divided into two groups: geriatric and non-geriatric. For this, age was coded as ‘aged 64 years or younger’ and ‘aged 65 years or older.’ Education level was coded as ‘education level of university or higher’ and ‘education level of high school or lower.’ The participants with an education level of university or above were considered as having a high education level. Marital status was coded as ‘married’ and ‘single or divorced.’ For categorization of income level, the official minimum wage in Turkey was considered as the reference value, and the level was coded as ‘income more than minimum wage’ and ’income less than minimum wage.’ Chronic disease status was coded as ’chronic disease’ and ‘no chronic disease.’ Social security status was coded as ‘social security’ and ’no social security.’ There was no significant discordance in the number of patients between the subgroups coded for the regression analyses. Patients with age of 64 years or less (non-geriatric) had a 2.6-fold higher probability for CAM use (95% CI [1.445–4.684]); patients with education level of university or higher (high education level) had a 1.54-fold higher probability for CAM use (95% CI [1.133–2.081]); patients with income more than the minimum wage had a 1.46-fold higher probability for CAM use (95% CI [1.072–1.993]); and the patients with presence of a chronic disease had a 1.57-fold higher probability for CAM use (95% CI [1.160–2.122]) (Table 5).

**Table 5:** Factors affecting the CAM use.

Factors	95% CI for EXP(B)				
	B	Exp(B)	Lower	Upper	Sig.
Being at the age of 64 years or younger	**0.956**	**2.601**	**1.445**	**4.684**	**0.001**
Being female	0.001	1.001	0.748	1.338	0.997
Having an education level of university or higher	**0.429**	**1.536**	**1.133**	**2.081**	**0.006**
Being a single or divorcee	0.156	1.168	0.855	1.596	0.329
Having an income more than minimum wage	**0.380**	**1.462**	**1.072**	**1.993**	**0.016**
Having a chronic disease	**0.451**	**1.569**	**1.160**	**2.122**	**0.003**
Having a social security	0.038	1.039	0.575	1.876	0.900

**Notes.**

sig, significance

## Discussion

In this descriptive study, we aimed to assess CAM usage in patients admitted to the ER of a tertiary hospital in Turkey. The frequency of CAM usage in our study was 47.3%, and only 18.7% of the patients reported that they were against CAM and were not interested in its use. There are different percentages of CAM usage reported in the literature, according to the targeted population. Koç & Çınarlı (2018) reported that the frequency of CAM use among patients in ERs in Turkey was 94%. In studies conducted on different diseases in Turkey, the frequency of CAM use in patients with cancer was 41.1% (Gözüm, Tezel & Koc, 2003), in patients with type 2 diabetes mellitus, was 36.7% (Yıldırım & Marakoğlu, 2018), and in infertile women, was 51% (Özkan, Karaca & Sarak, 2018). In studies conducted on patients in ERs, Waterbrook et al. (2010) reported the frequency of CAM usage as 54.7%, and Yates, Armour & Pena (2009) reported it as 56.1%. In a systematic review, Jatau et al. (2016) stated that the prevalence of CAM usage among patients in the ER ranged from 1.4% to 68.1%.

There may be various reasons attributed to the different reported rates of CAM use in the literature. The definition of CAM differs across studies. In some studies, praying or balneotherapy were defined as CAM methods. Because of this categorization, the reported frequency of CAM use may have been higher in some studies (Chui et al., 2014). We based our definition on the CAM regulations of the Turkish Ministry of Health (MoH) for determining the types of CAM methods in this research (Turkish Official Gazette, 2014). In the literature, different methods have been used to determine the frequency rates of CAM use among patients in ERs. Studies have evaluated current CAM use, CAM use in the past year or during lifetime. If a patient had used a CAM method even once, we listed it as ‘use’ in this study. Additionally, sociocultural differences, lifestyles, religion, and ethnic origin may have influenced the results.

The most commonly used CAM methods reported in our study were herbal therapy, massage, dietary supplements, and hijama. In the literature, it has been well documented that herbal therapy is a frequently preferred method among CAM users (Adib-Hajbaghery & Hoseinian, 2014; Arentz et al., 2014; Jatau et al., 2016). A multicenter study in Turkey revealed that the most commonly used CAM method among the Turkish population was herbal therapy (Şimşek et al., 2017). Consistent with our results, the study also reported a high rate of hijama use among the CAM methods. Similar to our results, Li et al. (2004) reported the most common CAM methods in their study to be herbal therapy, massage, and vitamins. The easy access to herbal cures, and the belief that it is completely natural and harmless increases the use of herbal treatments. Additionally, the fact that patients do not have sufficient information about drug-herbal therapy interactions may increase their rates of use. Also, we believe that the frequency of hijama use is high in our study, as a result of cultural differences and religious beliefs in Turkey.

Out of all the CAM users, only 17.9% (*n* = 79) stated that they had their CAM treatment done by a healthcare professional. 49.8% of these patients claimed to have completely benefited from their CAM treatment, and 45.3% reported to have been partially benefited from the treatment. It was a surprising finding, since despite the fact that most of the CAM procedures were not performed by a healthcare professional, there was a high level of satisfaction among patients with regard to their CAM treatment. In another study from Turkey, half of the CAM users reported that the treatment was performed by a healthcare professional (Okyay & Koçyiğit, 2018). The study was conducted on patients with pain and might not reflect the entire society (Arnold et al., 2000). In 2014, the Turkish MoH regulated the principles of CAM use and only certificated healthcare professionals were authorized to use CAM methods. Practitioners other than healthcare professionals are unlicensed and do not receive formal education programs in Turkey (Okyay & Koçyiğit, 2018), and should not administer CAM treatment. However, our results suggest that the regulations made by the MoH have not achieved this aim. From our results, we consider that most CAM treatments were performed outside of health institutions and hospitals, by non-healthcare professionals. This may lead to infections, organ failure, delays in treatment, and death among CAM users. Unfortunately, almost all CAM users consider that CAM methods are natural and harmless (Şimşek et al., 2017).

In our study, the main reason for using CAM was positive advice received from previous CAM users. The second most common reason was the avoidance of drug adverse effects. Other reasons for CAM use were curiosity, immune system strengthening, inadequacy of conventional medicine, suggested use by healthcare professionals, and because CAM methods were cheaper. Although variable rates were determined, similar reasons have been identified for the reasons behind CAM use in previous studies (Jatau et al., 2016; Şimşek et al., 2017). In another study conducted on patients in the ER, the reasons for CAM use were to feel better, to relax, to strengthen the immune system, to decrease anxiety, to try them, and because someone else used them and was satisfied (Koç & Çınarlı, 2018).

In this study, the main information source of the patients was relatives. This was followed by the internet, media, healthcare professionals, and books/journals. Koç & Çınarlı (2018) reported that the most important information sources for CAM were television, the internet, newspapers/journals, medical staff, and medical books/articles. Waterbrook et al. (2010) reported that the most common information source was friends, and only 8% of patients were informed by their physicians. The main reason for the discordance between the above-mentioned studies is methodologic differences. Koç & Çınarlı (2018) did not present ‘relatives and family’ as a source of information in their questionnaire form. Using relatives, friends, television, and the internet as a source of information may cause various problems. Patients may acquire inaccurate or incomplete information about CAM methods. Therefore, healthcare professionals should be educated on CAM methods and patients should be informed about CAM in health institutions.

Being aged 64 years or younger, having an education level of university or higher, having an income more than the minimum wage, and having a chronic disease were found to be associated with CAM use in the present study. In contrast, sex, marital status, and social security status have not been seen to be associated with CAM use. Different results demonstrated sociodemographic characteristics and CAM use links in the literature. Being a high school graduate or above (Kim et al., 2005), female sex (Gözüm, Tezel & Koc, 2003), being aged 65 years or over (Yates, Armour & Pena, 2009), and having a chronic disease (Jatau et al., 2016) were defined as factors associated with CAM use. Koç & Çınarlı (2018) found no associations between sociodemographic characteristics and CAM use. The differences between the above-mentioned results may depend on the different characteristics of the participants evaluated in the studies. Being in a developed or developing country, religion, culture, insurance systems, and beliefs may influence the results.

This study has some limitations. This was a single-center study conducted in the ER of a tertiary hospital. Traditional, socio-cultural, and economic differences within the country could not be evaluated; therefore, our data cannot be generalized. We excluded patients with poor general condition, which may have affected our results. We did not evaluate ER visits related to CAM use. Although the literature was taken into account in the development of the questionnaire, and a pre-test evaluation was performed, there is no gold standard for validation.

## Conclusion

Nearly half of the patients in our study had used CAM at least once in the past. The most frequently used CAM methods were herbal therapy, massage, dietary supplements, and hijama. Less than one-fifth of CAM users reported that a healthcare professional performed the CAM method. Being aged 64 years or younger, having an education level of university or higher, having an income more than minimum wage, and having a chronic disease were found to be associated with CAM use in this study. The main information source of the patients was relatives and a high proportion of the patients did not use healthcare professionals as a source of information. Therefore, patients have a potential to obtain incorrect or incomplete information about CAM methods. We consider that physicians should incorporate CAM use history in their patient assessments. Taking into account the rates of CAM use, health care professionals should provide accurate and unbiased information about CAM methods.

##  Supplemental Information

10.7717/peerj.7584/supp-1 Supplemental Information 1 Questionnaire form prepared by the researchers and used for collecting data

10.7717/peerj.7584/supp-2 Data S1 Raw data used for all statistical analysis

## Additional Information and Declarations

### Data Availability

The following information was supplied regarding data availability: Raw data is available as a Supplemental File.
