# Supplementary material for: Assessment of complementary and alternative medicine use among patients admitted to the emergency room: a descriptive study from a Turkish hospital
Source: PeerJ. 2019 Aug 20;7:e7584. doi: 10.7717/peerj.7584 (PMC6707338; doi:10.7717/peerj.7584)
Supplement: Supplemental Information 1 [file peerj-07-7584-s001.docx]

**Age:**

**Sex:**

**Level of education:**

1. Primary school or below b) Middle school c) High school d) University or above

**Income status:**

**Marital status:**

1. Married b) single c) divorced / widow

**Social security status:**

1. Yes b) No

**What is your reason for admitting to emergency room ?**

a. Musculoskeletal Diseases

b. Gastrointestinal system diseases

c. Respiratory system diseases

d. Cardiovascular system diseases

e. Neurological diseases

f. Urological diseases

g. Gynecological diseases

h. Addiction / psychiatric diseases

I. Other**…**

**What is the frequency of admitted to emergency room? (how many times in the last year)**

**Do you have chronic diseases?**

1. Yes b) No

**Do you use regular medication(s) for this chronic disease?**

1. Yes b) No

**What do you think about complementary / alternative medicine?**

a. I'm against these treatments and I'm not interested

b. I am not against these treatments

c. I am not against these treatments and I am interested

**Have you ever used one of the complementary / alternative medicine methods ?**

1. Yes b) No

14. If yes, which of the following methods of complementary / alternative medicine did you use? (You can select more than one option)

a. Acupuncture

b. Apitherapy (treatment of bee and bee products)

c. Herbal therapies

d. Food supplements (probiotics, fish oil, special diets)

e. Hypnosis / meditation

f. Homeopathy

g. Chiropractic

h. Cupping

I. Larvae / Leech

j. Hijama

k. Mesotherapy

l. Prolotherapy

m. Osteopathy

n. Ozone

o. Reflexology

p. Massage

q. Aromatherapy

r. Music therapy

s. Other...................................

**Was the complementary / alternative medicine method applied to you by a medical staff?**

1. Yes b) No

**Did you benefit from complementary / alternative medicine method?**

a) Yes

b. Partially

c. No

17. **What are your reasons for using complementary / alternative medicine method? (You can select more than one option)**

a. For medical staff recommended

b. Traditional medicine was inadequate

c. To avoid drug side effects

e. To strengthen the immune system

e. for receiving positive advice from a past user

f. to try

g. Because it is cheaper

**What are your sources of information about complementary / alternative medicine medicine? (You can select more than one option)**

a. Medical staff

b. Media (TV, newspaper)

c. Internet

d. Relatives, neighbors, friends, etc.

e. Book and journal
